# Biochemical and Biophysical Characterization of Carbonic Anhydrase VI from Human Milk and Saliva

**DOI:** 10.1007/s10930-022-10070-9

**Published:** 2022-08-10

**Authors:** Alma Yrjänäinen, Maarit S. Patrikainen, Latifeh Azizi, Martti E. E. Tolvanen, Mikko Laitaoja, Janne Jänis, Vesa P. Hytönen, Alessio Nocentini, Claudiu T. Supuran, Seppo Parkkila

**Affiliations:** 1 Faculty of Medicine and Health Technology, Tampere University, Tampere, Finland; 2 Department of Computing, University of Turku, Turku, Finland; 3 Department of Chemistry, University of Eastern Finland, Joensuu, Finland; 4 Fimlab Ltd, Tampere University Hospital, Tampere, Finland; 5 Neurofarba Department, Sezione di Chimica Farmaceutica, University of Florence, Florence, Italy

**Keywords:** CA6, CA VI, Mass spectrometry, Size exclusion chromatography, Glycosylation, Oligomerization

## Abstract

Carbonic anhydrases (CA, EC 4.2.1.1) catalyze the hydration of carbon dioxide and take part in many essential physiological processes. In humans, 15 CAs are characterized, including the only secreted isoenzyme CA VI. CA VI has been linked to specific processes in the mouth, namely bitter taste perception, dental caries, and maintenance of enamel pellicle, and implicated in several immunity-related phenomena. However, little is known of the mechanisms of the above. In this study, we characterized human CA VI purified from saliva and milk with biophysical methods and measured their enzyme activities and acetazolamide inhibition. Size-exclusion chromatography showed peaks of salivary and milk CA VI corresponding to hexameric state or larger at pH 7.5. At pH 5.0 the hexamer peaks dominated. SDS- PAGE of milk CA VI protein treated with a bifunctional crosslinker further confirmed that a majority of CA VI is oligomers of similar sizes in solution. Mass spectrometry experiments confirmed that both of the two putative N-glycosylation sites, Asn67 and Asn256, are heterogeneously glycosylated. The attached glycans in milk CA VI were di- and triantennary complex-type glycans, carrying both a core fucose and 1 to 2 additional fucose units, whereas the glycans in salivary CA VI were smaller, seemingly degraded forms of core fucosylated complex- or hybrid-type glycans. Mass spectrometry also verified the predicted signal peptide cleavage site and the terminal residue, Gln 18, being in pyroglutamate form. Thorough characterization of CA VI paves way to better understanding of the biological function of the protein.

**Supplementary Information:**

The online version contains supplementary material available at 10.1007/s10930-022-10070-9.

## Introduction

Carbonic anhydrases, CAs, are fundamental and ubiquitously expressed enzymes catalyzing the hydration of carbon dioxide [1]. CAs are among the fastest enzymes known (the turnover number or Kcat of some CA isoforms exceeds 1 × 10^6^ s^−1^) and take part in a remarkable range of physiological processes, such as acid–base and fluid balance, calcification, photosynthesis, respiration, metabolism, and cell growth [1, 2]. There are 15 isoforms of α-CAs that are characterized in humans [3]. Among them is CA VI, the only secreted isoenzyme, that is produced by serous acinar cells in parotid and submandibular glands and human mammary glands resulting in secretion into saliva and human milk, respectively [4, 5].

In 1981, CA VI (called Gustin at that time) was linked to taste function regulation [6]. *CA6* gene polymorphism (rs2274333 A/G) has been linked to bitter taste modality contributing to 6-n-propylthiouracil (PROP) taster status [7]. The alterations in bitter taste function are due to polymorphic changes in the bitter receptor gene (*TAS2R38*) and in the *CA6* gene, and to contributions from other still unknown factors [8]. Salivary CA VI functions on the tooth surface located in enamel pellicle [9] and is suggested to have a role in the natural defense systems against dental caries [10]. Immunoglobulin A (IgA) anti-CA VI autoantibodies are frequently seen in patients with long-term Sjögren’s Syndrome (SS) and antibodies to *SP1*, *CA6* and *PSP* gene products provide valuable markers for the diagnosis of primary and secondary SS [11, 12]. A unique form of murine CA VI, cytosolic CA VI-b, is an enzyme highly responsive to CHOP-dependent stress-inducible expression [13]. CA VI-b is suggested to be directly connected to the innate immune response by selectively inducing cytokine IL-12 production through protein arginine N-methyltransferase 5 (PRMT5) and regulating symmetric dimethylation at Arg-8 histone H3 dimethyl R8 (H3R8me2s) modification, independent of its CA activity [14].

Although milk and salivary CA VI have been studied parallelly with SDS-PAGE and Western blotting [15, 16], the oligomeric state of mature human CA VI with the full-length C-terminus has not been characterized. Our earlier study discovered a pentameric assembly of zebrafish pentraxin-CA VI [17], but in this case the pentamerization was assumed to be mediated by the pentraxin domain, similar to that observed for human C-reactive protein. The pentraxin domain is only present in non-mammalian vertebrate CA VI but absent from mammalian CA VI, so this result cannot be extrapolated to human CA VI. The protein produced for crystallization of human CA VI [18] behaved as a mixture of monomers and dimers in solution by SEC and as a dimer in the crystal structure. However, the recombinant protein was devoid of residues 291–308, outside of the catalytic domain, which are predicted to form an amphipathic alpha helix [17]. In a study of lacrimal sheep CA VI was also observed as a dimer in Western blotting [19]. The present study was conducted to provide additional information regarding the molecular weight and particle size of native human milk and salivary CA VI as well as to assess their post-translational modifications. In particular, we were interested in glycosylation, because one of the N-glycosylation sites in human CA VI is highly conserved across all vertebrate species [17]. This suggests it is essential to have a glycan at that particular position, and knowledge of the glycan structures would be needed for further studies of potential functional consequences. Furthermore, we prepared molecular models of glycosylated monomer and dimer forms of human CA VI.

## Materials and Methods

### Protein Isolation, Purification, and Analysis

CA VI protein was extracted from human breast milk donated by breastfeeding mothers at Tampere University Hospital (Pirkanmaa Hospital District, Tampere, Finland) and stored frozen at the hospital. The milk samples given to this study were anonymous, past their expiration date, and destined for destruction at the maternity clinic. The milk samples were thawed and pooled, and 1000 ml of milk was clarified by centrifugation (16,000 × *g*, 30 min at + 4 °C, Sorvall Lynx 4000, Thermo Fisher Scientific, MA, USA) and supernatant was collected and filtered using cellulose filter in a Büchner funnel by means of a vacuum. The clear flow-through was diluted to a volume of 5000 ml using 0.1 M Tris-SO_4_, 0.2 M Na_2_SO_4_ pH 8.7, to which 50 ml of 0.1 M Tris-SO_4_, 0.2 M Na_2_SO_4_, with protease inhibitor 0.2 M benzamidine, pH 8.7 was added, and subjected to affinity purification. The inhibitor affinity chromatography was performed by using p-aminomethylbenzenesulfonamide-agarose (Sigma-Aldrich, St. Louis, MO, USA). 12 ml of agarose was washed twice with H_2_O and twice with 0.1 M Tris-SO_4_, 0.2 M Na_2_SO_4_, pH 8.7 and centrifuged 85 × *g*, 4 min, each time. The diluted milk sample was incubated with washed p-aminomethylbenzenesulfonamide-agarose at room temperature (RT) for at least 1 h or at + 4 °C overnight. Wash and elution buffers for protein purification were prepared and the actual purification steps were performed according to the original protocol as described earlier [15].

Human saliva was collected from healthy volunteers (members of the Tampere research group, who gave their consent to use the saliva in this study) into an ice-cold centrifuge tube containing 2 ml of 0.1 M Tris-SO_4_ buffer containing 0.2 M Na_2_SO_4_, 0.2 M benzamidine, pH 8.7. For protein purification, 300 ml of pooled saliva were centrifuged (16,000 × g, 30 min) for removing extraneous material. Then, saliva supernatant was diluted with 0.1 M Tris-SO_4_ binding buffer containing 0.2 M Na_2_SO_4_, pH 8.7 to 1.5 L. Six ml of washed p-aminomethylbenzenesulfonamide–agarose was added and left on a magnetic stirrer at + 4 °C overnight. The purification steps were carried out as described above.

Protein purity was assessed with 12% SDS-PAGE under reducing conditions. The relative molecular mass of the protein was estimated using Precision Plus Protein™ Standards Dual Color (Bio-Rad Laboratories, Inc., Hercules, CA, USA) and the molecular weight marker, salivary and milk CA VI bands were visualized using the PageBlue™ Protein Staining Solution (Thermo Fisher Scientific). For further analyses, the elution buffer of both salivary CA VI and milk CA VI (50 mM Tris-SO_4_, 0.4 M NaN_3_, 1 mM benzamidine, 20% glycerol, pH 7.0) were changed to 50 mM Tris–HCl, pH 7.5 for enzyme activity measurements and size-exclusion chromatography and to 100 mM ammonium acetate for mass spectrometry. The protein concentrations were measured with a Thermo Scientific™ NanoDrop™ One Microvolume UV–Vis Spectrophotometer (Thermo Fisher Scientific) using 55,935 cm^−1^ M^−1^ as absorption coefficient, calculated for human CA VI without signal peptide by ProtParam (https://web.expasy.org/protparam/) [20].

An Applied Photophysics stopped-flow instrument was used for assaying the CA-catalyzed CO_2_ hydration activity [21]. The method was exactly as described previously [22] except that the inhibitor dilutions were done up to 0.5 nM.

### Analytical Size-Exclusion Chromatography (SEC)

The molecular weight of CA VI in solution was determined with analytical SEC using a high-performance liquid chromatography (HPLC) instrument (CBM-20A, Shimadzu Corporation, Kyoto, Japan) equipped with a UV–vis absorbance detector (SPD 20A, Shimadzu). The system control and data analysis were done using Lab Solution version 5.51 (Shimadzu Co.) software. For this purpose, two replicate samples of 50 µg of milk CA VI and salivary CA VI were applied through an autosampler onto a Superdex 200 5/150 GL column (GE Healthcare, Uppsala, Sweden) with a flow rate of 0.1 ml/min at a constant temperature of + 12 °C. The column was equilibrated in filtered 50 mM Tris–HCl, pH 7.5 which was used as a running buffer throughout the whole run. Similar samples were also run in 0.1 M Na-acetate buffer, pH 5.0, to give a total of eight runs for this experiment. Column calibration was performed by running standard proteins of the gel filtration marker kit (MWGF200, Sigma-Aldrich): carbonic anhydrase II 29 kDa, bovine serum albumin 66.7 kDa, alcohol dehydrogenase 150 kDa, and beta-amylase 200 kDa. Protein molecular weights were calculated using the standard curve based on the elution volumes of the standard proteins. Molecular weight of 33.57 kDa was used in calculating oligomeric state of CA VI, based on the protein sequence of UniProt P23280, CAH6_HUMAN [23] residues 18–308 (signal peptide excluded).

### Crosslinking reaction using DSS

To evaluate the oligomeric state in solution, a crosslinking experiment of milk CA VI was performed using disuccinimidyl suberate (DSS) (21,658, Thermo Fisher Scientific). The stock solution of DSS and the buffers were prepared according to the manufacturer’s instructions. For this, milk CA VI sample concentrations of 0, 0.25, 0.5, 0.75, 1, 1.5 and 2 mg/ml were prepared in the 100 mM Na_2_HPO_4_, 0.15 M NaCl at pH 7.5. The crosslinker DSS was added to each sample in 25- and 50-fold molar excess over protein of the crosslinker. The reaction was mixed carefully but gently and let them incubate at RT for 30 min. Next, the quenching buffer, 20 mM Tris–HCl pH 7.5, was added to stop the reaction. Samples were boiled five minutes in sample buffer containing SDS and reducing agent before analyzing them with SDS-PAGE, followed by staining using the PageBlue™ Staining Solution (Thermo Fisher Scientific). Molecular weights were estimated based on the MW marker in SDS-PAGE on a 12% gel, and ImageLab v5.2 MW analysis tool was used for densitometric assessment of relative quantities (Image Lab Software, RRID:SCR_014210). To double check the high-MW range of the crosslinked products, the same samples were also run on a 7.5% acrylamide gel.

### Liquid Chromatography – High-Resolution Mass Spectrometry (LC-HRMS)

Milk and salivary CA VI samples were digested with trypsin (T1426; Sigma-Aldrich) using acetonitrile–water (50:50, v/v) as solvent. The protein concentration was 1 µM in all the digestions and a trypsin-to-protein ratio of 1:50 (w/w) was used. Dithiothreitol (DTT) was added to the protein solutions at 100 µM concentration to reduce the disulfides. Each digestion was performed overnight at RT and stopped by adding 1 µl of formic acid, after which the solutions were stored at + 4 °C. Prior to the LC-HRMS experiments, acetonitrile was removed under vacuum. All the solvents used were of HPLC grade.

All LC-HRMS experiments were performed on a Dionex UltiMate 3000 RSLC liquid chromatography system (Thermo Fisher Scientific) connected to the high-resolution 12-T Bruker solariX XR Fourier transform ion cyclotron resonance (FTICR) mass spectrometer (Bruker Daltonik GmbH, Bremen, Germany). The peptides were separated on a Thermo Acclaim PepMap100 C18 column (75 µm i.d. × 15 cm, 3 µm) with an injection volume of 5 µl. The column oven temperature was set to 30 °C and a flow rate of 0.5 µl/min was used. The mobile phases were as follows; A: 100% H_2_O + 0.2% formic acid, B: 100% acetonitrile + 0.2% formic acid. For the LC separations, the following gradient used was: 1% B for 0–10 min, 1–40% B in 10–70 min (linear gradient), 85% B in 70–80 min, and 1% B for 80–90 min. The LC system was directly connected to the standard Bruker nanoelectrospray ion source, operated in positive-ion mode. The drying gas flow rate was set to 4 l/min, the nebulizer gas pressure was 0.3 bar, and the ion source temperature was 180 °C. Prior to the measurements, the instrument was calibrated externally using arginine clusters (0.1 mg/ml in MeCN/H_2_O). The mass range used was *m/z* 200–4000, and a single time- domain transient (1 Mword) was used for each spectrum over the entire LC–MS run. Ion accumulation time was set to 0.1 s for MS and 0.5 s for MS/MS measurements. The MS/MS measurements were performed automatically in a data-dependent fashion, based on the intensities and charge states of the detected ions. The fragmentation voltages were optimized based on the previous study [17]. The LC and MS instruments were controlled using Chromeleon 6.8 (Thermo Scientific), ftmsControl 2.2 and HyStar 4.2 (Bruker Daltonics) software. The chromatograms and the mass spectra were further processed and analyzed using Bruker DataAnalysis 4.4 and GPMAW 10.0a (Lighthouse Data, Odense, Denmark).

### Molecular Modelling

All molecular images and animations were created using ChimeraX (daily build 1.4.dev202202030703), developed by the UCSF Resource for Biocomputing, Visualization, and Informatics (San Francisco, California, USA), supported in part by the National Institutes of Health [24].

Theoretical models of glycosylated CA VI were created starting from PDB 3FE4 [18], chain B. In silico glycosylation was performed at GlyProt (http://www.glycosciences.de/modeling/glyprot/php/main.php) [25]. Glycans were taken from the associated Glycom-DB database of 3D oligosaccharide structures at http://www.glycosciences.de [26]. We display the closest matches to the largest glycans discovered in our work (underlined in Fig. 2d). For milk CA VI we used a core fucosylated triantennary glycan 8914 at position 256 and a core fucosylated biantennary glycan 8388 at position 67. For salivary CA VI, the same glycans were trimmed to leave the largest discovered salivary glycans.

Another glycosylated model was made based on the model of human CA VI in the AlphaFold Protein Structure Database (https://alphafold.ebi.ac.uk/entry/P23280, retrieved Jan 16th, 2022) [27]. Residues 1–17 corresponding to the signal peptide were removed, and the orientation of the C-terminal alpha helix was adjusted by manually rotating protein backbone bonds in the linker region 280–282. Glycans were added in silico following the procedure described above for 3FE4.

A glycosylated dimer model of the full sequence of human CA VI was created by superimposing one copy of the glycosylated AlphaFold model on each of the chains in 3FE4, using the MatchMaker tool of ChimeraX and a pruning cutoff of 3.0 Å. Residues 32–278, or as much of the catalytic domain as is present in 3FE4, were used for the superimpositions.

The amphipathic helix region 289–308 was saved as a PDB file from the AlphaFold model in ChimeraX and submitted into protein–protein docking using ZDOCK v. 3.0.2. (https://zdock.umassmed.edu/) [28]. The helix model was used as both input molecule 1 and input molecule 2, and no residues were selected or blocked as docking targets. An antiparallel helix pair was selected for further use from the 10 ZDOCK predictions.

A hexameric model of the full sequence of mature human CA VI was made by joining three copies of dimeric AlphaFold models (without glycans) using our antiparallel helix dimer model as a scaffold to lock the helix orientations between the middle dimer and peripheral dimers. The two helices left free in the ends were brought next to each other by manually rotating protein backbone bonds in the linker region 280–282 of several monomers.

## Results

### Enzyme Activity and Acetazolamide Inhibition

Carbonate dehydratase activity was measured kinetically without and with a clinically used CA inhibitor, acetazolamide (5-acetamido-1,3,4-thiadiazole-2-sulfonamide), with results shown in Table 1. The k_cat_ values for CA VI enzymes from three different sources are within a very narrow range, and fall between those measured for highly active human CA isoforms CA I and CA II [29]. CA VI purified from milk had a slightly smaller k_cat_ value than the one purified from saliva or recombinantly produced. All samples of human CA VI showed slightly less activity than the pentameric zebrafish CA VI [17]. CA VI from all three sources were highly inhibitable by acetazolamide, with nanomolar KI values.

**Table 1:** Activity and inhibition measurements of human CA VI compared to other CA isozymes

Sample	k_cat_ (s^−1^)	KI (acetazolamide) (nM)
Recombinant CA VI^a^	3.4 × 10^5^	11
Salivary CA VI	3.3 × 10^5^	16
Milk CA VI	2.3 × 10^5^	23
Human CA I^b^	2.0 × 10^5^	250
Human CA II^b^	1.4 × 10^6^	12
Zebrafish CA VI^c^	8.9 × 10^5^	5

^a^Human recombinant isozyme [36]

^b^Human CA I and human CA II[29]

^c^Zebrafish recombinant enzyme[17]

### Molecular Weight Determination using Analytical Size-Exclusion Chromatography

**Fig. 1:**
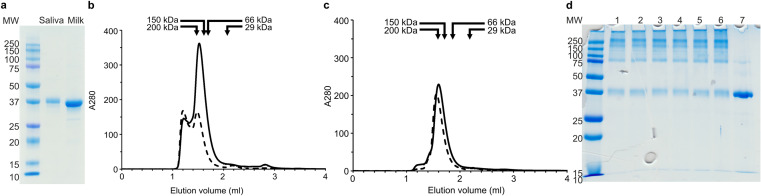
Determination of the oligomeric state of human CA VI by SEC and crosslinking **a** SDS-PAGE of purified CA VI, with molecular weight markers on the left and 2.5 µg protein of each CA VI sample. Salivary CA VI, ~ 40 kDa, and milk CA VI, ~ 38 kDa. **b** and **c** Size-exclusion chromatography of salivary CA VI (solid line) and milk CA VI (dashed line). Vertical axes shows A280 × 1000. The samples were run in 50 mM Tris–HCl, pH 7.5 (**b**) or 0.1 M Na-acetate buffer, pH 5.0 (**c**). **d** SDS-PAGE on a 12.5% acrylamide gel of crosslinked samples, with 50-fold molar excess of DSS (over protein) and increasing concentrations of milk CA VI on lanes 1 to 6: 0.25, 0.5, 0.75, 1, 1.5 and 2.0 mg/ml, respectively. MW markers are on the left, and lane 7 shows the protein without DSS treatment. 2.5 µg of CA VI was loaded on each of lanes 1 to 7

We purified CA VI protein from human saliva and milk using inhibitor affinity chromatography for experiments of its characterization. Initial evaluation of the size of the purified protein was performed using SDS-PAGE, yielding bands slightly above the MW calculated from the protein sequence, as expected with two N-glycans (~ 2–3 kDa each) (Fig. 1a). To observe the oligomerization state of the protein sample, SEC was used. Figure 1b and c show the elution patterns of salivary and milk CA VI proteins at pH 7.5 (Fig. 1b) and pH 5.0 (Fig. 1c), and the results are summarized in Table 2. When run at pH 7.5, we see two major peaks for both salivary and milk CA VI, peak 1 at ~ 470 kDa and ~ 490 kDa, respectively, and peak 2 at 177 kDa and 202 kDa, respectively. For milk CA VI peak 1 and peak 2 were approximately equal in intensity (Fig. 1b, solid line), whereas for salivary CA VI peak 1 is less than half of peak 2 in intensity (Fig. 1b, dashed line). In pH 5.0 the largest peaks nearly disappear (Fig. 1c), and the main peaks are at 214 kDa and 243 kDa for salivary and milk CA VI, respectively. There is a tiny peak near 2.2 ml for milk CA VI in both runs, but this corresponds to only 21 kDa and is therefore an impurity or degradation product, and one peak at even smaller MW for salivary CA VI. No peaks corresponding to monomers were seen for either salivary or milk CA VI in either pH value. Peak 1 is calculated to represent oligomers of 14 or 15 units, whereas peak 2 is calculated to be pentamers to heptamers as shown in Table 2.

To validate the SEC results and confirm the proximity of the protein subunits in solution, we used a short, homobifunctional crosslinker DSS. While untreated CA VI purified from saliva and milk appears mostly as a monomer in denaturing SDS-PAGE, the milk CA VI protein sample treated with DSS crosslinker appeared at multiple molecular sizes. At the end of the crosslinking experiment, major species of approximately 34 to 37 kDa, 69 to 78 kDa, 146 to 192 kDa, and 224 to > 250 kDa were identified, corresponding to milk CA VI monomers, dimers, pentamers, and hexamers (Table 3). In each replicate experiment the monomers seem most abundant, followed by hexamers and pentamers. Because the crosslinking reaction is not necessarily complete, the original distribution of oligomers in solution may contain more of the larger oligomers, as indicated by the SEC results. Very faint bands in Fig. 1c, between dimer and pentamer bands, are compatible with the sizes of trimers and tetramers, but they are not shown in Table 3 because they were not consistently quantitated by the gel scanning software. It is notable that even the CA VI sample without the crosslinking reagent shows a small amount (~ 10%) of dimer. To check that there no high-MW CA VI oligomers which did not penetrate in the 12% running gel (Fig. 1 d shows some large-MW residue at the top of the gel), we reran the same samples in a 7.5% gel, with essentially identical results (Supplementary Fig. S1).

**Table 2:** Size-exclusion chromatography of CA VI

Sample	Elution volume (ml)	MW (kDa)	Putative oligomeric state^a^
Salivary CA VI in Tris pH 7.5, peak 1	1.219	467	~ 14-mer
Peak 2	1.521	177	Penta-/hexamer
Salivary CA VI in Na-Acetate pH 5.0	1.598	214	Hexamer
Milk CA VI in Tris pH 7.5, peak 1	1.204	490	~ 15-mer
Peak 2	1.480	202	Hexamer
Milk CA VI in Na-Acetate pH 5.0	1.559	243	Heptamer

^a^Calculated assuming monomer MW of 33.57 kDa

**Table 3:** Molecular weights and percentages of crosslinked human milk CA VI bands shown in Fig. 1c

Lane	CA VI (mg/ml)	Monomer		Dimer		Tetra- or pentamer		Hexamer	
		MW (kDa)	%	MW (kDa)	%	MW (kDa)	%	MW (kDa)	%
1	0.25	37.22	43.05	77.79	16.14	192.25	14.13	> 250.00	22.30
2	0.5	36.62	28.43	73.83	9.88	171.74	10.25	> 250.00	12.84
3	0.75	35.81	40.44	71.90	19.43	158.11	15.18	244.14	20.32
4	1	35.39	43.11	70.50	17.03	153.47	13.18	230.55	23.53
5	1.5	34.95	42.50	69.74	16.87	146.80	12.68	225.00	20.41
6	2	34.97	45.79	69.61	19.04	148.21	12.14	223.82	20.79
7	Native CA VI	33.98	88.25	68.84	9.67				

### Characterization of Post-Translational Modifications in Milk and Salivary CA VI

Preliminary mass analyses of the intact milk CA VI with LC-FTICR MS showed a heterogeneous signal of protein with an approximate mass of 38 kDa (data not shown). CA VI contains two putative N-glycosylation sites, Asn67 and Asn256 (both comprising an NxT consensus motif), present on the surface of the protein [5]. Since the polypeptide chain of mature CA VI is 33.57 kDa in size, glycosylation occurring on both sites would account for the additional mass observed. In the salivary CA VI, the main protein signals were obtained at around the same retention times as the milk protein, however the peaks remained unresolved, suggesting more heterogeneity in the protein structure (data not shown). In addition, minor protein signals, e.g., at 29.1 and 55.9 kDa, were also observed upon the intact mass measurements, suggesting the presence of some other minor proteins in the purified samples. For the further structural characterization, both salivary and milk CA VI proteins were subjected to in-solution trypsin digestion followed by the LC-FTICR MS/MS analysis.

**Fig. 2:**
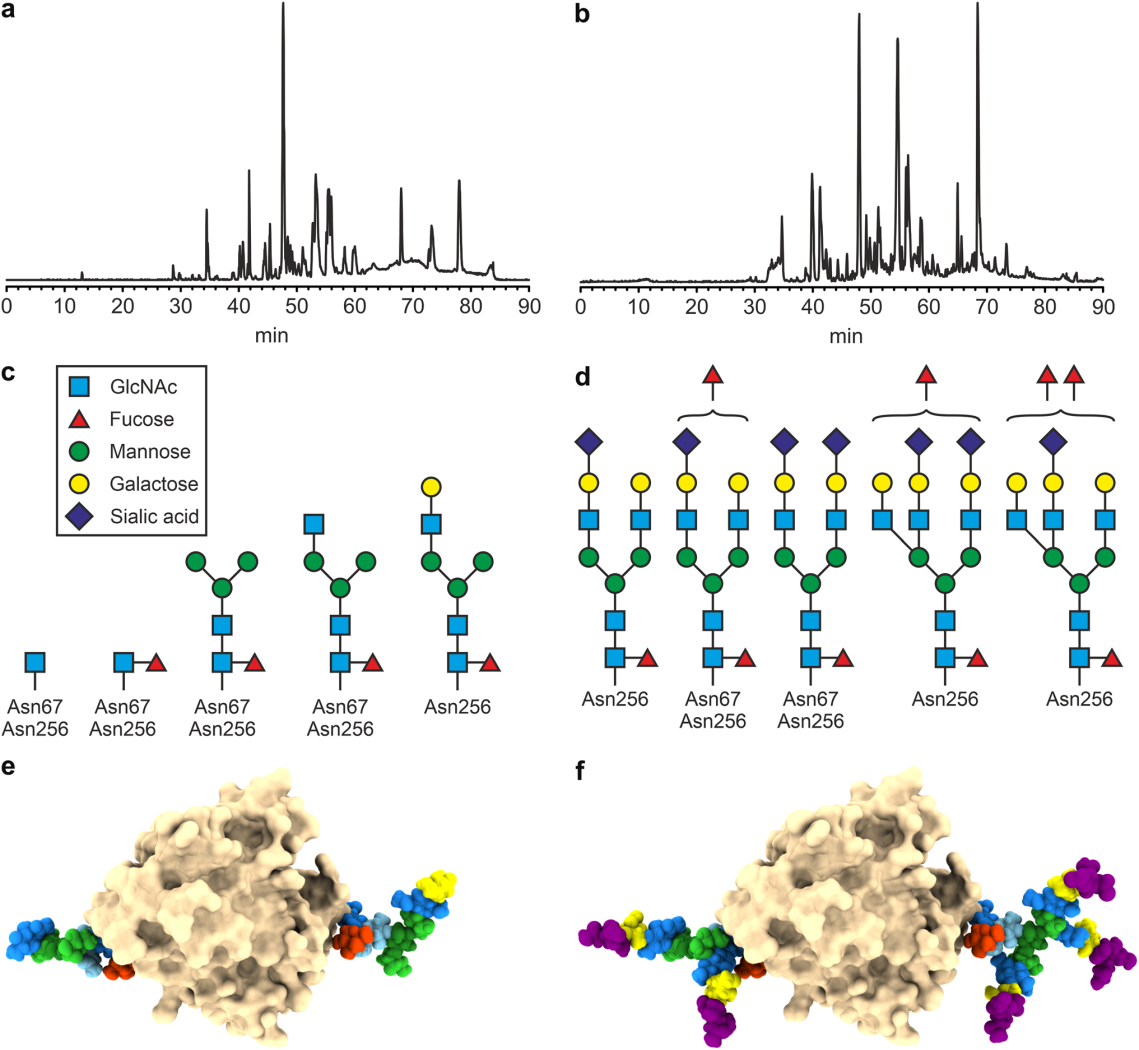
Mass spectrometry analysis of human salivary and milk CA VI **a** and **b** Total ion chromatograms (TIC) for the LC–MS/MS runs of the tryptic digests of CA VI from saliva (**a**) and milk (**b**). **c** and **d** Glycan structures discovered by MS/MS in glycopeptides of CA VI from saliva (**c**) and milk (**d**). **e** and **f** Molecular models with representations of the largest discovered glycans of salivary CA VI (**e**) and milk CA VI (**f**). White/grey: human CA VI (PDB 3FE4, oriented with the active facing away from the viewer); glycan on the right is at Asn256 and glycan on the left is at Asn67. Monosaccharides are colored as in panels **c** and **d**, with a lighter blue distinguishing the second GlcNAc (from the reducing end) in the chain, and a darker green distinguishing the β-Man in the branchpoint of the glycan antennae

Figure 2a and b show the total ion chromatograms (TIC) for the LC–MS/MS runs of the tryptic digests of both protein samples. From the tryptic digest of the salivary CA VI, a total of 192 peptides were found, 102 of which were confidently identified based on the monoisotopic masses and the MS/MS fragmentation patterns. Similarly, from the tryptic digest of the milk CA VI, 224 peptides were found, 142 of which were uniquely identified. In both samples, most of the identified peptides originated from CA VI (75 and 61 for the salivary and milk CA VI, respectively). Several amino acid variants were also discovered, adding to the sample heterogeneity. In the milk samples, two natural CA VI variants, M68L and S90G, were observed. In the saliva samples, a double variant M68L + G70A and a triple variant M68L + G70A + S90G were also found. Moreover, several other (minor) proteins were observed in both samples such as amylase in the salivary sample and fatty acid synthase, different caseins, and also CA II in the milk sample. Table 4 shows more details of the detected proteins, CA VI sequence variants and their corresponding single nucleotide polymorphisms. Both milk and saliva samples used in CA VI purification were pooled from multiple donors, and as the allele frequencies for the corresponding three SNPs range from 12 to 58% in Finnish population (data from Ensembl release 104, http://May2021.archive.ensembl.org/Homo_sapiens/Gene/Variation_Gene/Table?db=core;g=ENSG00000131686;r = 1:8,945,867–8,975,092), these variants could be expected.

**Table 4:** Proteins identified from the tryptic digests of salivary and milk CA VI samples

Sample origin	Protein	Uniprot ID	Number of identified peptides
Saliva	Carbonic anhydrase VI	P23280	75
	- M68L (dbSNP:rs2274328)^a^		
	- G70A (dbSNP:rs2274329)^a^		
	- S90G (dbSNP:rs2274333)^a^		
	Amylase	P04075	18
	Mucin-7	Q8TAX7	2
	Immunoglobulin A		
Milk	Carbonic anhydrase VI	P23280	61
	- M68L (dbSNP:rs2274328)^a^		
	- S90G (dbSNP:rs2274333) ^a^		
	Fatty acid synthase	P49327	46
	Beta-casein	P05814	7
	Kappa-kasein	P07498	6
	Carbonic anhydrase II	P00918	6
	Alpha-casein	P47710	5
	- A117V (dbSNP:rs10030475)^a^		
	Galectin-3-binding protein	Q08380	5
	Clusterin	P10909	4
	Angiopoietin-related protein 4	Q9BY76	2

^a^Variants from dbSNP database, https://www.ncbi.nlm.nih.gov/snp/

**Fig. 3:**
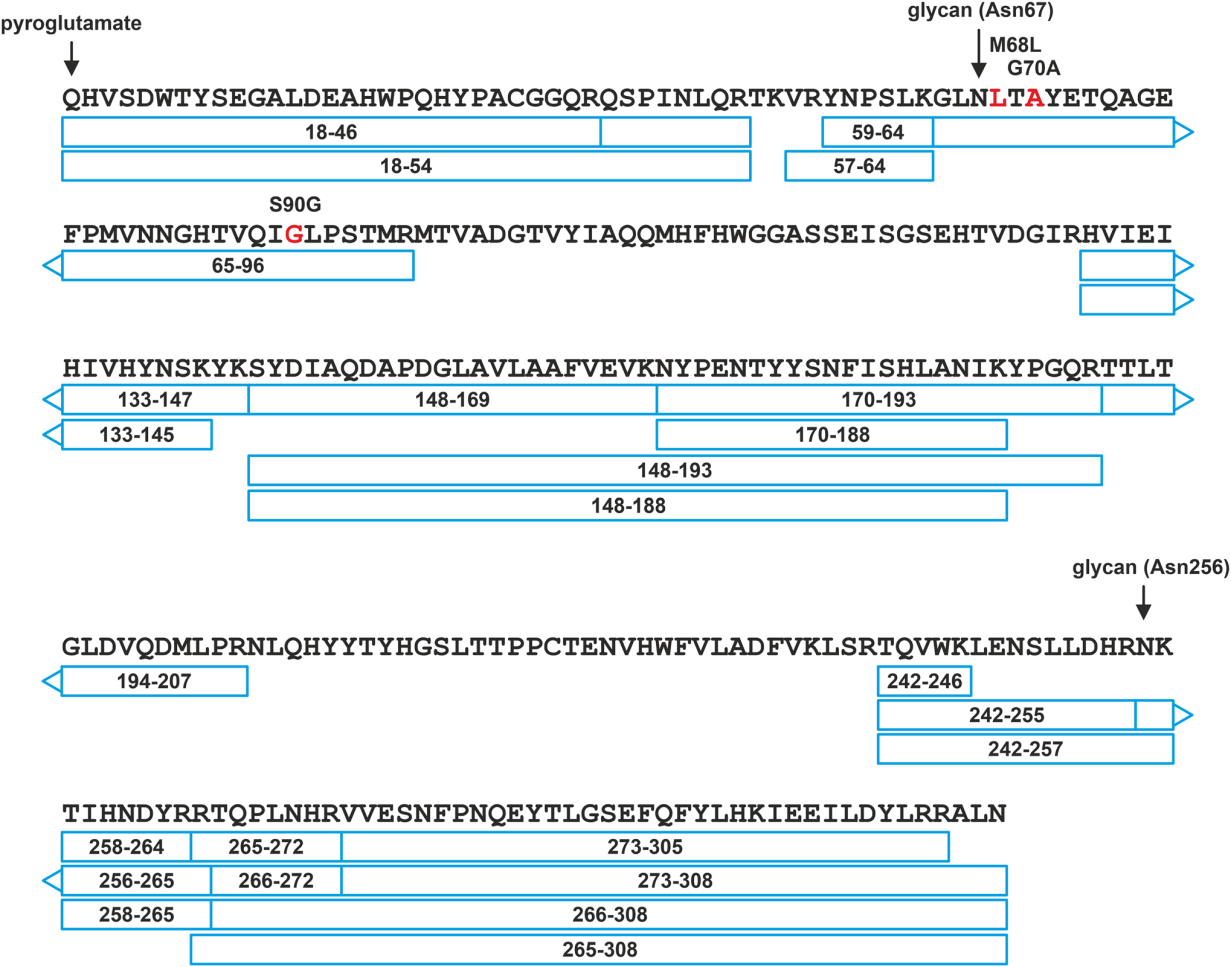
Sequence coverage (75%) obtained for human salivary CA VI based on MS/MS-identified peptides numbering according to UniProt P23280. N-terminal pyroglutamate (pQ18) formation was observed in the peptides. Glycosylation sites (Asn67 and Asn265) marked with arrows and observed sequence variant sites with red color

**Fig. 4:**
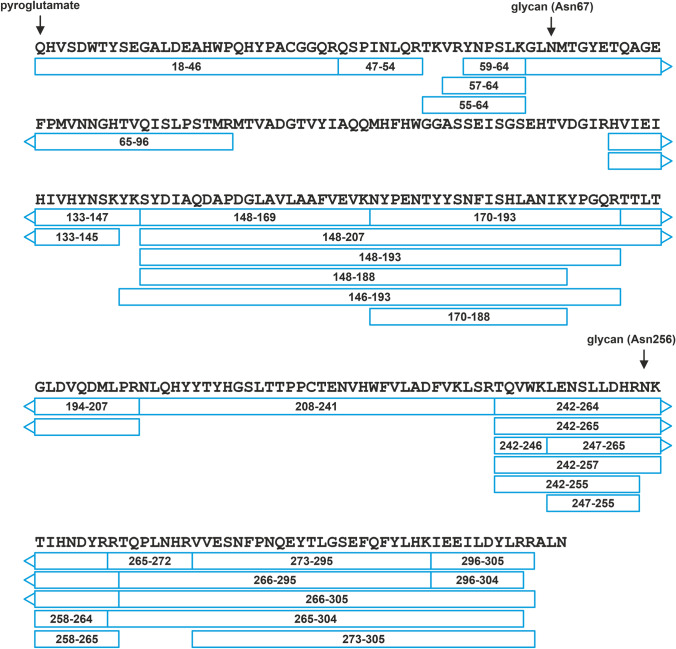
Sequence coverage (86%) obtained for human milk CA VI based on MS/MS-identified peptides. Numbering according to UniProt P23280. N-terminal pyroglutamate (pQ18) formation was observed in the peptides. Glycosylation sites (Asn67 and Asn265) are marked with arrows. Variants M68L and S90G as indicated in Fig. 3 were also observed

In both samples, an N-terminal pyroglutamate formation was observed, and the N-terminal residue (Gln18) confirms the predicted signal peptide cleavage site (UniProt P23280). In the salivary protein sample, three other minor proteins (i.e., amylase, and trace amounts of immunoglobulin A and mucin) were identified, consistent with the sample origin. In the milk sample, a relatively high number of peptides originating from fatty acid synthase were observed. In addition, trace amounts of alpha-casein, beta-casein, and kappa-casein were identified, which are typical proteins found in milk as well. A small number of peptides resulting from the trypsin auto-proteolysis was also observed. The tryptic peptides covered 75% and 86% of the sequence for saliva and milk CA VI, respectively (Figs. 3 and 4).

**Fig. 5:**
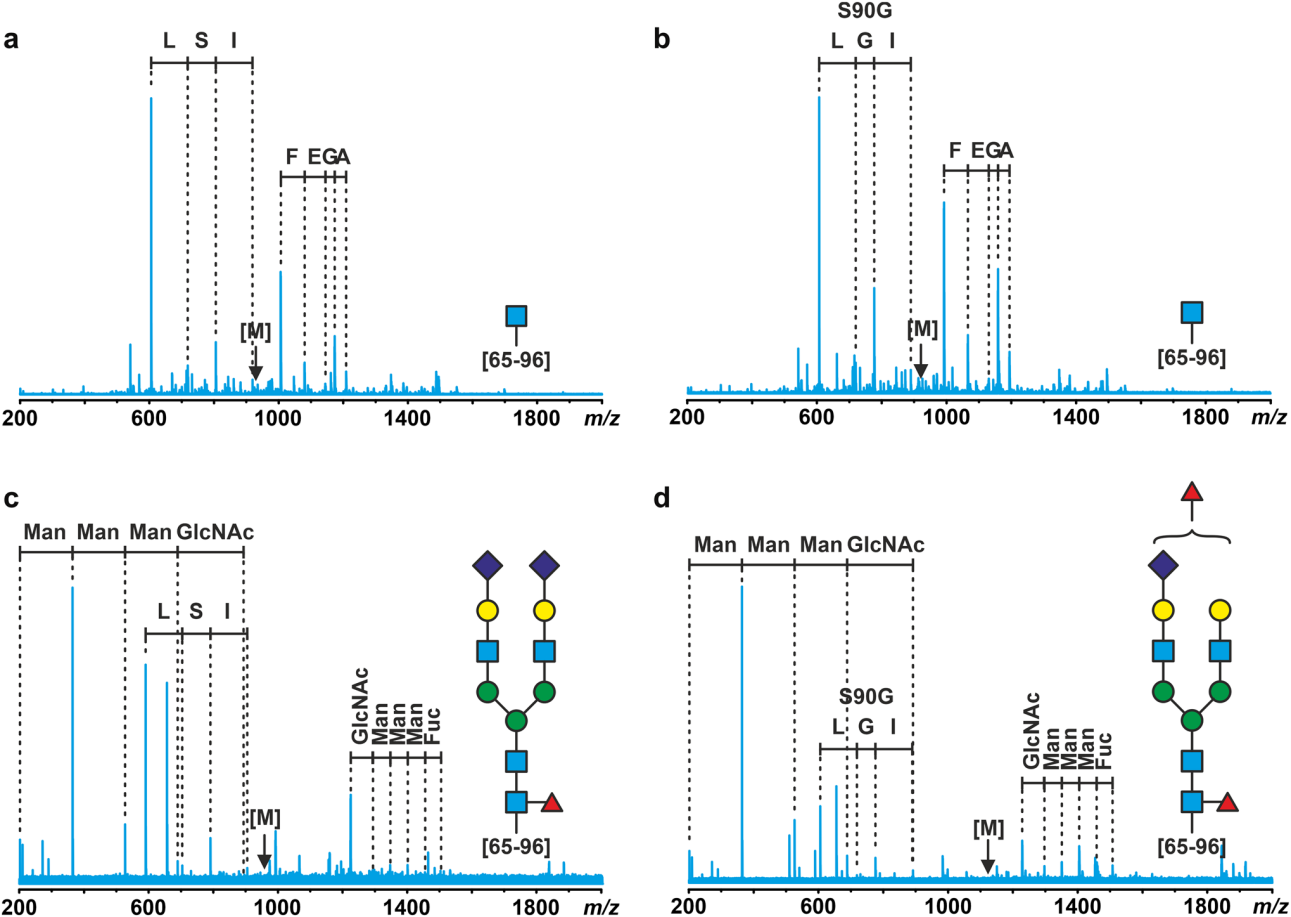
MS/MS spectra of glycopeptides in human salivary and milk CA VI. Peptide sequence identified as GLNMTGYETQAGEFPMVNNGHTVQISLPSTMR [65–96] (fragmentation sites underlined). **a** and **b** Fragmentation patterns observed for peptides from salivary CA VI containing a single GlcNAc residue, **b** also contains an S90G variant. **c** and **d** Peptides observed in milk CA VI containing large complex-type glycan structures, **d** also contains an S90G variant

Among the identified tryptic peptides, 24 and 18 glycopeptides were observed in the milk and salivary CA VI samples, respectively. The MS/MS experiments further confirmed the peptide sequences and the glycan structures of these peptides. Representative fragmentation patterns are shown in Fig. 5. The glycopeptides confirmed that both putative N-glycosylation sites in CA VI, Asn67 and Asn256, were heterogeneously glycosylated. The attached glycans in the salivary CA VI were core-fucosylated, seemingly degraded forms of complex- or hybrid-type glycans. In contrast, the glycans in the milk CA VI were found to be much larger, complex-type (di- and triantennary) glycans, carrying both the core fucose and 1 to 2 additional fucose units (Fig. 2c and 2d). A visualization of the largest glycans discovered in salivary CA VI are shown in Fig. 2e. For milk CA VI, di- and triantennary oligosaccharides similar to the largest ones found in this study are shown in Fig. 2f. The colors of the monosaccharide units follow Fig. 2c and d. The model of Fig. 2f is additionally shown in an animation (online resource 1) which shows views from all directions around the model.

### Molecular Models

**Fig. 6:**
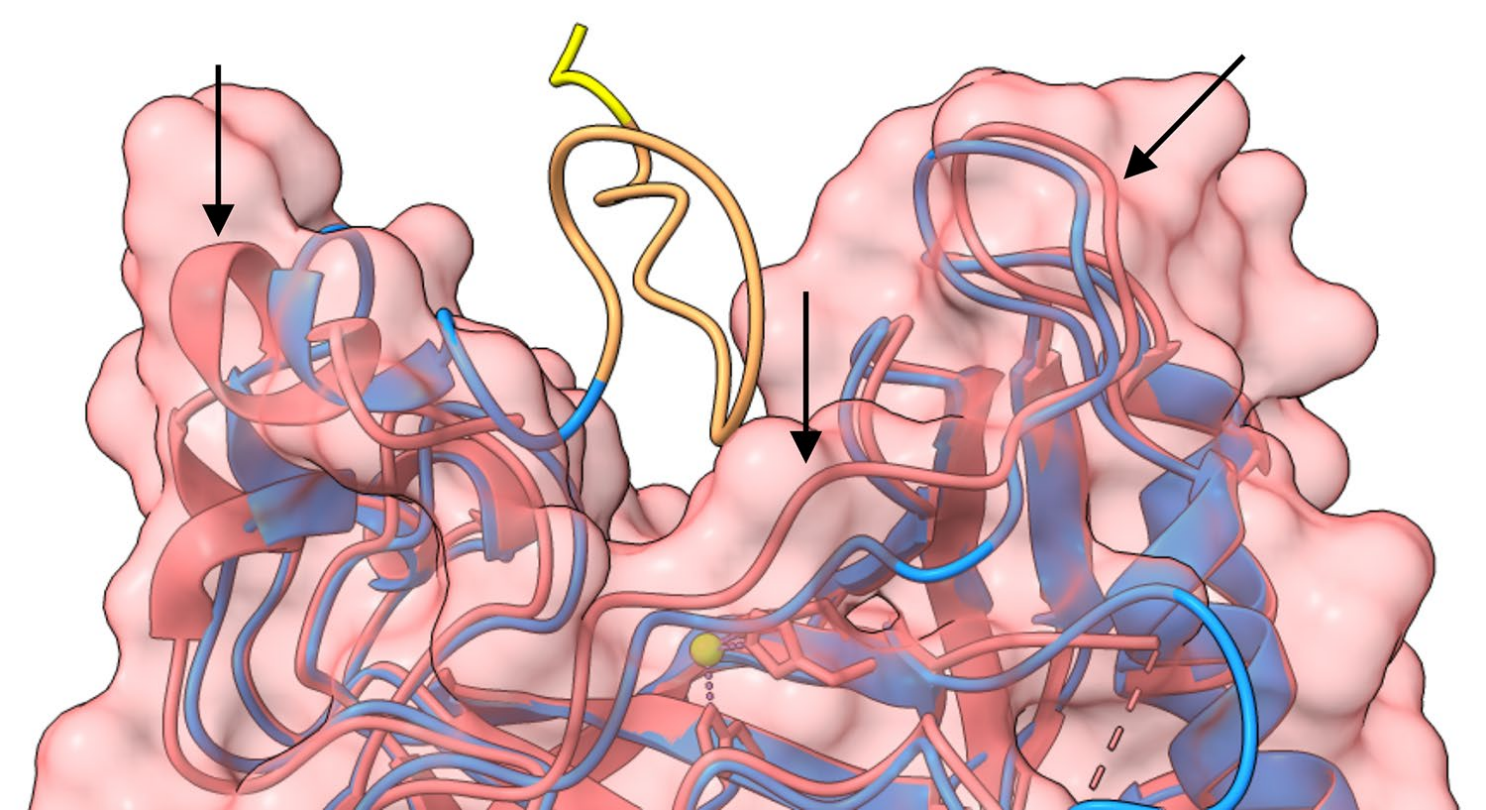
Comparison of human CA VI models from PDB and AlphaFold. Blue, AlphaFold model (signal peptide 1–17 not shown). Pink, PDB 3FE4. Pink surface corresponds to 3FE4, and arrows indicate the regions with most difference between 3FE4 and the AlphaFold model in the vicinity of the active site. Brown ribbon, outside the surface, is for AlphaFold model residues 21–31 which are not seen in 3FE4, and yellow ribbon is residues 18–20 which are not even present in the construct that was made for crystallization of 3FE4 (18–19 missing, and 20 replaced by Met instead of Val) (Color figure online)

We compared the AlphaFold model for human CA VI to PDB 3FE4 chain A, using 3D superimposition of the visible residues 32–278. RMSD between 234 pruned atom pairs is 0.855 Å (across all 243 pairs: 1.095 Å). Figure 6 highlights the most dissimilar regions around the active site between the two models, on three sides of the region 20- 31 which, even if present in the crystallized recombinant protein, is not modelled in 3FE4 due to disorder. The hypothetical conformation for 20–31 in the AlphaFold model is shown in brown, and additional residues 18 and 19 are in yellow. These residues were not even part of the protein construct used in crystallization of 3FE4. We can note that the region occupied by the “invisible” part of the sequence is wider but shallower in 3FE4 (pink) than in the AlphaFold model (blue).

The signal peptide was included in the full AlphaFold model. Residues 1–20 form a long, extended chain which is ranked to be of very low confidence in the AlphaFold prediction [per-residue confidence score (pLDDT) < 50 in a scale of 0 to 100]. Figure 6 is made with the signal peptide 1–17 hidden. Because of the context, resides 18–20 in the AlphaFold model extend away from the folded domain, but this would be less likely in a model of the mature protein.

**Fig. 7:**
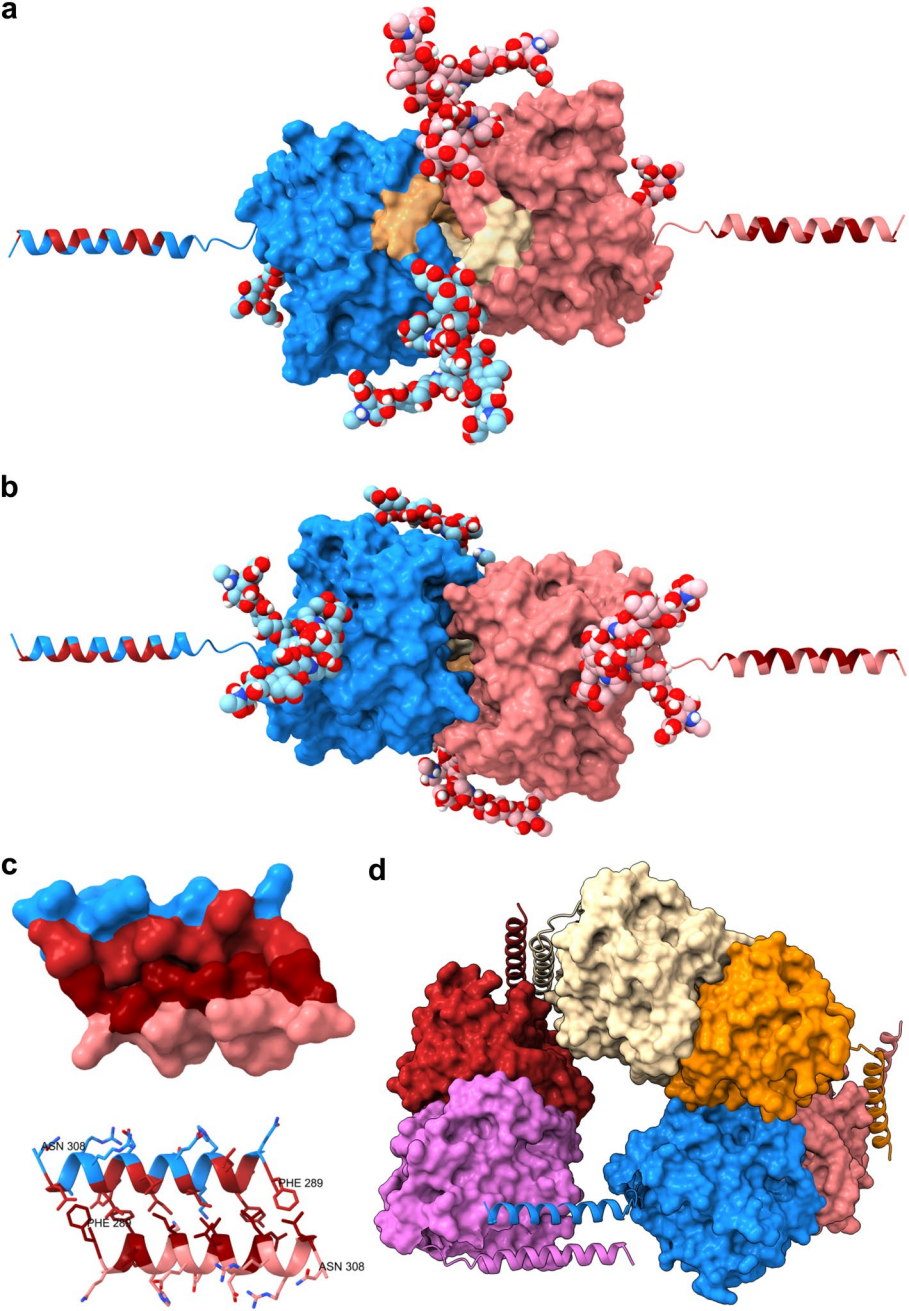
Molecular models of full-length human CA VI. **a** and **b** Two views from opposite sides of a glycosylated dimer model based on the AlphaFold model of human CA VI. White and khaki regions in **a** are residues 18–31 which are not visible in PDB 3FE4. Pink and blue glycans are attached to the pink and blue monomers, respectively. Glycosylation site Asn256 is at the foreground in **a**, whereas the site at Asn67 is at the foreground in **b**. Hydrophobic residues in the C-terminal helices are indicated in red-brown. **c** Two representations of a model for dimerization of the amphipathic C-terminal helix, residues 290–308, with coloring as in panels **a and b**. **d** Hypothetical ring arrangement of a hexamerix CA VI complex (Color figure online)

We also created new molecular models based on the AlphaFold model. Figure 7a shows a dimeric model of human CA VI, based on superimposition of two AlphaFold monomers (18–308) on the dimer structure of 3FE4. Residues 18–19 clash with the other monomer but no attempt was made to fix that. One can observe how the probably flexible residues 18–31 (in khaki and white) are capable of filling a gap seen in the dimer interface of 3FE4, as predicted by Pilka et al. [18].

Figure 7a additionally demonstrates that the glycosylation site at Asn256, which is highly conserved among vertebrate CA VI sequences [17] is located near the dimer interface. Figure 7b shows a view rotated 180° horizontally relative to Fig. 7a. The glycosylation site at Asn67, which bears smaller glycans than Asn256 by our MS results, and which is not conserved in vertebrates, is located nearly opposite to Asn256 and not close to the dimer interface, seen at the foreground in Fig. 7b.

The CA VI monomers are bound with the active sites facing each other in the dimer. In Fig. 7b it can be seen that there is just a small opening in the dimer interface (near the midpoint of the image) which would provide access to the active sites for substrates and products (CO_2_ and bicarbonate being the largest species).

We have predicted the C-terminal region 289–308 to be folded as an amphipathic helix [17], and this is supported by the AlphaFold model. In Fig. 7a and b hydrophobic residues are colored in red-brown, with a clear distribution on one side of the helix. Figure 7c shows a model for dimerization of two of those helices. We produced ten helix dimer models by docking two copies of the helical segment 289–308 dissected from the AlphaFold model. No residues were chosen to be included in or excluded from the binding interface in docking. All resulting ten models had the hydrophobic sides of the helices facing each other. In six of the models alpha helices were parallel and in four antiparallel. One of the antiparallel models is shown in Fig. 7c, resembling a short, antiparallel coiled-coil.

We suggest that the formation of oligomers larger than dimers requires both face-to-face binding (as seen in PDB 3FE4) and helix-to-helix binding (as in Fig. 7c). To visualize one such hypothetical oligomer we created a chain of six copies of human CA VI by joining three dimers of the AlphaFold model with two helix-to-helix interactions. To test if the terminal helices of our hexamer chain can be brought together, to form a non-covalent cycle, backbone bonds in the linker region 280–283 between the CA domain and the helix were rotated. Figure 7d presents the result of this experiment, in which the red and white helices near the top of the image come from the loose ends of the initial chain.

## Discussion

Our SEC data of native milk and salivary CA VI and SDS-PAGE results of crosslinked milk CA VI indicate a that human CA VI with a full-length C-terminus forms oligomers larger than the previously observed dimers [18] (Fig. 1, Supplementary Fig. S1, and Tables 2 and 3). Because of heterogeneous glycosylation and no knowledge of the relative amounts of different glycoforms, it is challenging to estimate precisely the degree of oligomerization. Furthermore, MW estimates of oligomers larger than largest standard proteins is less reliable, as minor differences in the slope of the calculated trend line are accentuated. We also consider it possible that glycoproteins move anomalously in SEC because of the carbohydrate-based column material (dextran/agarose) which may interact with the glycans. Still, our consensus estimate from all of our experiments is that the major form of human CA VI in solution would be hexamers and larger, up to 14-to-15-mers at pH 7.5.

Decreasing the pH from 7.5 to 5.0 in SEC analyses dissociates the largest complexes (or makes their half-lifes shorter), so that the sample looks nearly homogeneous. Interestingly, the calculated MW of both salivary and milk CA VI increases by 20% in the runs at pH 5.0, from the same samples and on the same day, which could be caused by a difference in the interaction between column material and the glycans of CA VI. Another interpretation would be a transition from pentamer majority to hexamer majority, but our structural hypothesis of oligomerization makes hexamers more likely, which would mean attributing the changes in apparent MW to technical reasons.

Other human α-CAs have not been reported to be higher oligomers than dimers [30–32]. In case of zebrafish CA VI, the pentraxin domain explains the observed pentamerization, but this domain is not present in mammalian CA VI [17]. However, mammalian CA VI does possess a C-terminal extension of ~ 30 to 40 residues containing a moderately conserved region predicted to form an amphipathic helix [17], which distinguishes CA VI from other mammalian α-CA isoforms. We postulate that this unique feature is the basis for the observed higher oligomerization in human CA VI by allowing the formation of multimers of dimers through contacts between the hydrophobic sides of two amphipathic helices.

We observed no oligomers smaller than pentamers to heptamers in SEC, which would suggest that there is a stabilizing factor at those oligomer sizes, which we would postulate to be ring formation of CA VI protomers. In order to explore the possibility of oligomers in a ring shape, we created a hypothetical model of a hexamer of human CA VI (Fig. 7d). We started from a hypothesis of antiparallel coiled-coil-like contacts between the C-terminal amphipathic helices, which allow creating a linear chain of CA VI dimers to give more room for movement of the dimeric CA domains. We cannot exclude the possibility of parallel helix contacts either, or a combination of antiparallel and parallel contacts in a chain of dimers, but in our hands such combinations were harder to manipulate due to steric hindrance. The model we show only proves that a chain of three CA VI dimers can be arranged into a ring shape, but we do not claim the model to represent the most likely or only way in which cyclic oligomers could form, or that it proves cyclic oligomers to exist. There are several unrestricted residues between the helical and CA domains which can be easily rotated to allow many conformations. Cyclic octamers etc. would also be feasible, but a chain of four monomers does not allow a cycle to be built while keeping the face-to-face contacts. Furthermore, odd-numbered oligomers could be formed if an unpaired monomer is added in the end of a chain of dimers.

However, our interpretation is that the face-to-face interaction, with its larger surface area, is stronger than the helix- to-helix interaction, making the odd-numbered oligomers less likely under our hypotheses of CA VI oligomer formation.

The glycosylation site at Asn256, which is conserved in most vertebrate species, has a special location at the rim of the active site cavity. In the context of the dimer observed in PDB 3FE4, this site is near the dimer interface. It can be speculated that the glycans could interact with the other half of the dimer, stabilizing the dimer. This could explain the differences seen in activity and inhibition results of Table 1. The larger glycans of CA VI isolated from milk could be more stabilizing than the smaller glycans of CA VI isolated from saliva, translating into a decreased rate of dimer dissociation and slower access to the active site through the small opening seen in Fig. 7b. The inhibitor acetazolamide is larger than the substrates, explaining the slightly larger effect on inhibition than activity. The recombinant CA VI is produced in bacteria [36], in a form containing the signal peptide sequence, which could not form face-to-face dimers, and it is non-glycosylated. This form has the highest activity and highest susceptibility to inhibition.

Previously, CA VI purified from human saliva has been shown to contain a small amount of deglycosylated protein in addition to a major fraction which was similar in size to CA VI purified from milk in SDS-PAGE [5]. The smaller oligosaccharides observed in salivary CA VI in this study (Fig. 2c) must be degradation products of larger glycans, because the biosynthesis of all N-glycans starts with a tetradecasaccharide precursor. The smallest sugar moieties (only GlcNAc or Fuc-GlcNAc) can be explained by digestion with endo-beta-N-acetylglucosaminidases, which are known to be present in human saliva [33]. The relative shortness of antennae in the longer oligosaccharides of salivary CA VI could be explained by exoglycosidase action, either from oral microflora or human secreted enzymes, as multiple glycosidase activities are known to be present in human oral cavity [34, 35]. The hepta- and octasaccharides of Fig. 2c could also result from lower activities of glycosyl transferases in salivary glands, not just glycosidase degradation.

The complex-type glycans of milk CA VI are intriguing in that we discovered di- and tri-fucosylated forms. In addition to the core fucose, the branches contain up to two other fucoses, which could be e.g. β1-3 linked to antennary GlcNAc units or β1-2 linked to Gal units, based on known glycosyltransferase activities in the mammary gland [37]. More detailed knowledge of the glycan structures would allow further hypotheses of potential interactions with membrane lectins, but this awaits further studies.

## Supplementary Information

Below is the link to the electronic supplementary material.

Supplementary file1 (MP4 6828 kb)

Supplementary file2 (PDF 81 kb)

## Abbreviations

SEC: Size-exclusion chromatography
MS: Mass spectrometry
LC: Liquid chromatography
HRMS: High-resolution mass spectrometry
CA: Carbonic anhydrase
DSS: Disuccinimidyl suberate
LC-FTICR: Liquid chromatography Fourier transform ion cyclotron resonance
