# Supplementary material for: Biochemical and Biophysical Characterization of Carbonic Anhydrase VI from Human Milk and Saliva
Source: Protein J. 2022 Aug 10;41(4-5):489–503. doi: 10.1007/s10930-022-10070-9 (PMC9464147; doi:10.1007/s10930-022-10070-9)
Supplement: Supplementary file 2 — Supplementary file2 (PDF 81 kb) [file 10930_2022_10070_MOESM2_ESM.pdf]

Supplementary information for

Biochemical and Biophysical Characterization of Carbonic Anhydrase VI from Human Milk and Saliva

Alma Yrjänäinen[1], Maarit S. Patrikainen[1,*], Latifeh Azizi[1], Martti E.E. Tolvanen[2], Mikko Laitaoja[3], Janne Jänis[3], Vesa P. Hytönen[1,4], Alessio Nocentini[5], Claudiu T. Supuran[5], Seppo Parkkila[1,4]

[1]Faculty of Medicine and Health Technology, Tampere University, Tampere, Finland
[2]Department of Computing, University of Turku, Turku, Finland
[3]Department of Chemistry, University of Eastern Finland, Joensuu, Finland
[4]Fimlab Ltd, Tampere University Hospital, Tampere, Finland
[5]Neurofarba Department, Sezione di Chimica Farmaceutica, University of Florence, Italy
*Corresponding author: maarit.patrikainen@tuni.fi

A video file of the molecular model of Fig. 2f is provided as Online Resource 1 showing human CA VI glycosylated *in silico* with the glycans similar to the largest ones observed by MS in this study. The model is rotated around vertical and horizontal axes to provide views from all directions.

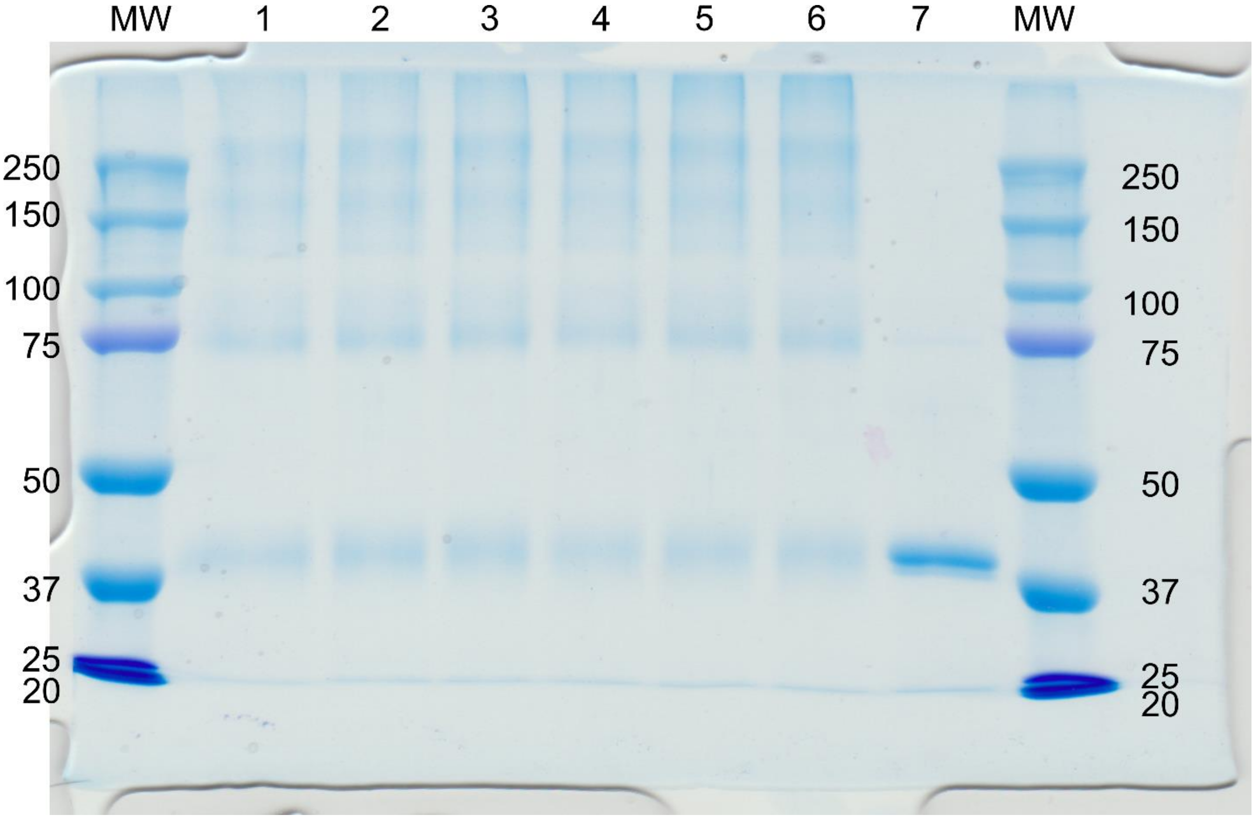

Supplementary Fig. S1 Determination of the oligomeric state of human CA VI by crosslinking. SDS-PAGE on a 7.5% acrylamide gel of crosslinked samples (same samples as in Fig. 1d), with 50 × molar excess of DSS (over protein) and increasing concentrations of milk CA VI on lanes 1 to 6: 0.25, 0.5, 0.75, 1, 1.5 and 2.0 mg/ml, respectively. MW markers are on the left and on the right, and lane 7 shows the protein without DSS treatment. 2.5 µg of CA VI was loaded on each of lanes 1 to 7
